# The Influence of Hippotherapy on the Body Posture in a Sitting Position among Children with Cerebral Palsy

**DOI:** 10.3390/ijerph17186846

**Published:** 2020-09-19

**Authors:** Ewelina Matusiak-Wieczorek, Elzbieta Dziankowska-Zaborszczyk, Marek Synder, Andrzej Borowski

**Affiliations:** 1 Sports Medicine Institute, Social and Preventive Medicine Department, Medical University of Lodz, 92-213 Lodz, Poland; 2 Epidemiology and Biostatistics Institute, Social and Preventive Medicine Department, Medical University of Lodz, 90-752 Lodz, Poland; elzbieta.dziankowska-zaborszczyk@umed.lodz.pl; 3 Orthopedics Department, Medical University of Lodz, 92-213 Lodz, Poland; marek.synder@umed.lodz.pl (M.S.); andrzej.borowski@umed.lodz.pl (A.B.)

**Keywords:** cerebral palsy, hippotherapy, postural control, balance

## Abstract

The purpose of this study was to assess the influence of hippotherapy (therapy with horses) on posture and body function among children with cerebral palsy. A case–control study included forty-five children aged 6–12 years, classified as Gross Motor Function Classification System (GMFCS) level I or II, with spastic diplegia or hemiplegia. The participants were randomly divided into three groups: study I (n = 15), study II (n = 15) and control (n = 15). The children from the study groups attended 30min hippotherapy sessions for 12 consecutive weeks, twice (study group I) or once (study group II) a week. The Sitting Assessment Scale (SAS) was used. A comparison of SAS showed an improvement in almost all the assessed categories among the children who participated in hippotherapy. In study group I, statistically significant differences were noted in the assessment of head position control, arm function (in both cases, *p* = 0.012) and trunk control (*p* = 0.005) and in study group II in the assessment of trunk control (*p* = 0.028). Hippotherapy has a positive influence on the body posture and function of individual body parts in a sitting position among children with cerebral palsy.

## 1. Introduction

Cerebral palsy is a disorder of movement, muscle tone and posture caused by an incorrect transmission of signals between the central nervous system and the muscles. This imbalance leads to increased activity in the muscles, impacting posture and walking patterns; this in turn results in limitation of daily activities and independence [1,2,3,4,5]. For this reason, the therapeutic process involves a number of methods and procedures that improve the motor function of those children. Apart from traditional physiotherapy, other forms of rehabilitation are sought to complement the treatment process. One of them may by hippotherapy. Hippotherapy is a therapy in which during riding and exercising on the back of a horse, the rider receives impulses from the horse which stimulate the work of his or her sensory, neuromotor and cognitive systems. Numerous studies confirm the beneficial influence of hippotherapy on the postural control, balance, gross motor functions and functional performance among children with cerebral palsy [6,7,8,9]. A detailed review of previous studies on the effects of hippotherapy on children with cerebral palsy is presented in the Supplementary Table S1.

During the hippotherapy session, a child sits on the horse’s back and tries to maintain an appropriate rider’s position while the horse is walking. With every minute, the horse sends many impulses to the rider, thus the child is stimulated to react and keep the position instead of falling down [10].

There is a need to conduct studies to better understand and evaluate the beneficial effects of hippotherapy on various disorders occurring in many diseases, that is why the purpose of this study was to assess the influence of hippotherapy on a child’s posture and functions of individual body parts according to the type of cerebral palsy, level of Gross Motor Function Classification System (GMFCS) and child’s age.

## 2. Materials and Methods 

Forty-five children aged 6–12 years with spastic diplegia or hemiplegia, classified as Gross Motor Function Classification System (GMFCS) level I or II were included in this study. All of them were able to understand and perform simple tasks. Hippotherapy was contraindicated in those who underwent an orthopedic, neurological surgery in the previous six months, and individuals who were unable to understand and perform tasks were excluded from the study.

The participants were randomly divided into three groups: study group I (n = 15), study group II (n = 15) and the control group (n = 15). Detailed characteristics of the participants are presented in Table 1.

The legal guardians were informed about the study and asked to give their prior written consent. The study was approved by the Bioethics Committee of the Medical University of Lodz, RNN/169/11/KE.

The children from the study groups participated in 30min hippotherapy sessions twice (study group I) or once (study group II) weekly for 12 consecutive weeks. The sessions were individualized according to each child’s needs and abilities. They took place in an indoor arena (10 × 30 m) and were conducted by a qualified therapeutic team. The horse walked along the arena walls from the right to the left for 15 min. During the first few laps in both directions, the child sat on the horse’s back and tried to maintain a proper rider position only, while the therapist gave verbal instructions and manipulated the child’s pelvis to provide support, if necessary. When the child adapted to the situation, the therapist presented exercises which the child had to perform, first when the horse was standing and then during walking. Those tasks were the following: to lean forward and touch the horse’s right ear with the left hand (and vice versa), to raise the upper limbs straightened to the front, then move them to the sides and rotate the trunk to the right and left, to put the hands on the back of the head, keeping the elbows wide apart and maintain this position for the whole lap. The child was supervised to perform the exercises correctly and to maintain the proper rider position. The final part of the ride, both on the right and left side, consisted of a few laps, during which the child, as at the beginning, had to sit in the correct position without doing any exercises.

All the children from the study groups were present at each session. The children from the control group did not undergo hippotherapy.

In order to assess the children’s posture and function of individual body parts, the Sitting Assessment Scale (SAS) was used, in accordance with the instructions provided by Ulla Myhr [11]. Unfortunately, the assessment was not filmed because the children’s legal guardians did not give their consent. That is why an assistant (who was a blinded examiner) was engaged in the study. The assistant sat at a table in front of the child and specified the order of tasks. The main investigator stood at a fixed distance from the side of the study station, made observations of individual body parts according to the SAS instruction and entered scores into the SAS evaluation sheet.

The child had to perform various tasks. Each of them took up to five minutes. During that time, the child repeated a task, while the investigator assessed the position and function of the head, trunk, feet, arms and hands using a four-point scale. Within 12 weeks, all the children participated in the above mentioned intervention four times. In the study groups, the first assessment was made prior to the first hippotherapy session, while in the control group it was performed after the inclusion of the child in the study. The next assessments were carried out every four weeks. The obtained information was analyzed with the STATISTICA package (StatSoft, Inc., Krakow, Poland, version 13, www.statsoft.com).

### Statistical Analysis

In order to compare changes in the scores received by each group when assessing the control of the position and function of individual body parts between the first and the last examination, Wilcoxon’s test was used. The significance level was assumed to be α = 0.05 and the differences were statistically significant for *p* ≤ 0.05.

The collected data also allowed for the distribution of changes in the assessment of body posture, taking into account the type of cerebral palsy, GMFCS level, and the child’s age. Fisher’s exact test was used to assess the significance of differences in the frequency of improvement in the groups. Every increase in the score on the SAS scale was considered as improvement. The significance level was assumed to be α = 0.05 and the differences were statistically significant for *p* ≤ 0.05.

## 3. Results

When comparing the results from the SAS scale obtained during the first and the last examination, it was noticed that the children from study group I improved in almost every category (except for foot control) (Table 2). Statistically significant differences were noted in the assessment of head position control, arm function (in both cases, *p* = 0.012) and trunk control (*p* = 0.005). At the end of the study, more than half of the children presented correct head position control, less than 50% had good control of arm function and over 70% gained three or four points for trunk control. 

In study group II, an improvement was observed in all the assessed categories (Table 2). However, statistically significant differences were noted only in the assessment of trunk control (*p* = 0.028). At the end of the study, over half of the examined children showed very good control of trunk position.

In the control group, an improvement was observed only in the control of trunk position and hand function (Table 2). However, those differences were not statistically significant. 

During the 12 weeks of the study, an improvement in body posture was most commonly observed in study group I, among children with hemiplegia. In study group II and the control group, the improvement was not so visible, both among children with diplegia and hemiplegia (Table 3). Statistically significant differences were shown only during comparison of the assessment of body posture between study group I and the control group (*p* = 0.001) and study group II and the control group (*p* = 0.051), in the children with hemiplegia. 

Changes in the SAS scale body posture assessment due to the children’s level of the GMFCS are presented in Table 4. At baseline, there were no statistically significant differences between the SAS ratings for different GMFCS levels. During the 12 weeks of the study, an improvement in body posture was most commonly observed in study group I, among children from the first level of GMFCS. At the end of the study, statistically significant differences were observed when comparing the assessment of body posture in the children from the first level of the GMFCS between study group I and the control group (*p* = 0.001) and study group I and study group II (*p* = 0.030). 

During the 12 weeks of the study, an improvement in body posture was most commonly observed in study group I, among 6−7-year-old children. Changes in the SAS scale body posture assessment due to the children’s age are presented in Table 5. Statistically significant differences were noticed between study group I and the control group (*p* = 0.000) and between study group II and the control group (*p* = 0.022), when comparing the assessment of body posture among the younger children (6−7 years old). 

## 4. Discussion

The term hippotherapy refers to treatment strategies based on the use of horse movement for improving postural control, balance and general function or mobility [10,12,13]. The main assumption in our study was that hippotherapy helps to improve body posture. As a result of observations and analyses, it turned out that in both study groups, the control of position and function of almost every assessed body part improved. In study group I, that had hippotherapy twice a week, statistically significant changes were seen in the assessment of head and arm control, while among the children from study group II, in trunk control. In Shurtleff et al.’s studies, among children who underwent hippotherapy, anterior–posterior translation of the head and spine decreased, which may suggest a better head and trunk stability. The researchers also observed an improvement in the function of the upper extremities [14,15]. In a study by Ionatamishvili et al., a reduction of involuntary movements of the head, trunk or extremities and a decrease in muscle tone, which improved motor functions among children with cerebral palsy, were presented [16]. Increases in strength, balance and muscle tone, leading to a better coordination of the movement of the upper and lower trunk, were also noticed in older publications, describing studies involving a smaller number of participants and applying hippotherapy or therapeutic horseback riding [17,18,19].

In our study, we assessed the effects of hippotherapy on postural control in children with diplegia and hemiplegia. We observed a significant improvement among those with hemiplegia when comparing study group I or study group II with the control group. 

In the study of Ionatamishvili et al., which included patients not only with a spastic type of cerebral palsy, the authors observed that effective motor activity improved after hippotherapy mostly in children with hyperkinetic rather than spastic cerebral palsy [16]. The reports presented above may suggest that equine-assisted therapy offers more considerable benefits to children with milder types of the disease.

The analysis of the data performed in our study showed that the statistically significant improvement of body posture as a result of hippotherapy occurred only among the children classified as GMFCS level I. At this point, it is worth mentioning that children classified as GMFCS level I and II already have quite good postural control, which could have contributed to not obtaining statistically significant results in the group of children classified as GMFCS level II. Positive results related to gross motor function and balance, not only for children with level I–III but also for those classified as GMFCS level IV, were presented in the publication by Kwon et al. [9]. In contrast, Hamill et al. pointed to the lack of improvement in the postural control in the sitting position among cerebral palsy children who underwent hippotherapy [20]. However, it has to be mentioned that this study included only three children and all of them were classified as GMFCS level V.

In our study, we examined the distribution of postural changes caused by hippotherapy related to the age of the children. In order to perform a statistical analysis, we divided the children into younger (6−7 years old) and older (8−12 years old) groups. When comparing study groups I or II with the control group, a significant improvement was always observed in the younger participants. To support our results, we can mention that Bertoti also observed more considerable benefits of therapeutic horseback riding among younger children [18].

Despite the increased interest in the topic of the influence of hippotherapy on children with cerebral palsy, the optimal frequency of riding which would bring the greatest benefits has not been determined yet. This is the reason why we decided to signalize this problem in our study and suggest investigating this in the future. We had two study groups in which the children received hippotherapy for 12 or 24 sessions. When comparing changes in the body posture during 12 weeks of hippotherapy we noticed an improvement in both groups. However, statistically significant improvement was more often observed among children who underwent hippotherapy twice a week. Results that are quite similar to those we obtained may be found in the study by Moraes et al. They applied hippotherapy twice weekly for 12 weeks and compared its effects on the postural balance after 12 and 24 sessions. They concluded that 24 sessions improved balance more effectively than 12 sessions [7].

The main limitation of the study is that it is difficult to estimate the impact of hippotherapy alone on the obtained results, because the children also participated in traditional rehabilitation, which, for obvious reasons, could not be interrupted for the duration of the study. The small study sample size might decrease the statistical power of the obtained results. Moreover, the lack of recordings of the intervention made it impossible to analyze it multiple times. Additionally, there may have been a risk of bias due to the study tool used.

However, we would like to emphasize that the comparison and equalization of results of hippotherapy in relation to the cerebral palsy type, GMFCS level or children’s age are difficult. That is why further research should be conducted in a homogeneous group of participants with the use of objective and standardized tools.

## 5. Conclusions

Hippotherapy could have a positive impact on the sitting body posture in children with cerebral palsy. The improvement of body posture that may be observed includes a better control of position and function of the head, trunk and upper extremities. As far as the child’s age, type of cerebral palsy or GMFCS level are concerned, hippotherapy may offer more benefits to younger children with milder forms of the disease, classified with higher levels of the Gross Motor Function Classification System.

## Figures and Tables

**Table 1:** Characteristics of the study participants.

	Study Group I (n = 15)	Study Group II (n = 15)	Control Group (n = 15)
**gender**	6 girls, 9 boys	7 girls, 8 boys	7 girls, 8 boys
**type of CP ^#^**	3 diplegia, 12 hemiplegia	2 diplegia, 13 hemiplegia	5 diplegia, 10 hemiplegia
**level of GMFCS ^#^**	10 level I, 5 level II	12 level I, 3 level II	7 level I, 8 level II
**range of age**	6−12	6−11	6−12
**mean age**	7.93 years (SD 2.6)	7.60 years (SD 1.84)	8.13 years (SD 2.56)

^#^ CP-cerebral palsy; GMFCS-Gross Motor Function Classification System

**Table 2:** Comparison of the average values of points in the Sitting Assessment Scale (SAS), for the study groups (SG) and the control group (CG), during the 12 weeks of the study.

SAS Evaluation (Points) (Mean ± SD)	Assessments in Consecutive Weeks				
	“0”	“4”	“8”	“12”	Difference ”12”–“0”
Head SG I	2.73 ± 0.88	2.93 ± 0.88	3.20 ± 0.77	3.40 ± 0.74	0.67 ± 0.72
Head SG II	3.73 ± 0.59	3.80 ± 0.56	3.93 ± 0.26	4.00 ± 0.00	0.27 ± 0.59
Head CG	3.60 ± 0.74	3.60 ± 0.74	3.60 ± 0.74	3.60 ± 0.74	0.00 ± 0.00
Trunk SG I	2.13 ± 0.99	2.53 ± 0.74	2.80 ± 1.01	3.00 ± 0.93	0.87 ± 0.74
Trunk SG II	2.93 ± 0.96	3.27 ± 0.70	3.40 ± 0.74	3.47 ± 0.64	0.53 ± 0.74
Trunk CG	2.93 ± 0.70	3.00 ± 0.76	3.07 ± 0.70	3.13 ± 0.64	0.20 ± 0.41
Foot SG I	1.47 ± 0.83	1.47 ± 0.83	1.47 ± 0.83	1.47 ± 0.83	0.00 ± 0.00
Foot SG II	2.53 ± 1.25	2.60 ± 1.18	2.67 ± 1.11	2.73 ± 1.16	0.20 ± 0.41
Foot CG	2.53 ± 1.19	2.53 ± 1.19	2.53 ± 1.19	2.53 ± 1.19	0.00 ± 0.00
Arm SG I	2.27 ± 0.96	2.27 ± 0.96	2.53 ± 0.83	2.80 ± 0.86	0.53 ± 0.52
Arm SG II	3.33 ± 1.05	3.33 ± 1.05	3.40 ± 0.91	3.53 ± 0.74	0.20 ± 0.41
Arm CG	3.00 ± 0.85	3.00 ± 0.85	3.00 ± 0.85	3.00 ± 0.85	0.00 ± 0.00
Hand SG I	2.33 ± 0.98	2.33 ± 0.98	2.40 ± 0.99	2.47 ± 0.92	0.13 ± 0.35
Hand SG II	3.47 ± 0.92	3.53 ± 0.74	3.53 ± 0.74	3.53 ± 0.74	0.07 ± 0.26
Hand CG	2.80 ± 0.77	2.80 ± 0.77	2.80 ± 0.77	2.87 ± 0.74	0.07 ± 0.26
TOTAL SG I	10.93 ± 3.97	11.53 ± 3.74	12.40 ± 3.70	13.13 ± 3.46	2.20 ± 1.42
TOTAL SG II	15.93 ± 4.17	16.53 ± 3.50	16.93 ± 3.24	17.27 ± 2.76	1.33 ± 0.76
TOTAL CG	14.87 ± 3.27	14.93 ± 3.35	15.00 ± 3.30	15.13 ± 3.36	0.27 ± 0.46

**Table 3:** Changes in the SAS scale rating for the type of CP.

Type of CP	Study Group I				Study Group II				Control Group				Differences between Groups *p* ^#^
	No Improvement		Improvement		No Improvement		Improvement		No Improvement		Improvement		
	(n)	(%)	(n)	(%)	(n)	(%)	(n)	(%)	(n)	(%)	(n)	(%)	
**diplegia**	**1**	33.33	2	66.67	1	50.00	1	50.00	3	60.00	2	40.00	(a)-(b) *p* = 0.600 (a)-(c) *p* = 0.429 (b)-(c) *p* = 0.571
**hemiplegia**	1	8.33	11	91.67	5	38.46	8	61.54	8	80.00	2	20.00	(a)-(b) *p* = 0.087 (a)-(c) ***p* =** 0.001 (b)-(c) ***p* =** 0.051
**diplegia vs. hemiplegia** ***p***	*p* = 0.344				***p*** = 0.514				***p*** = 0.330				

^#^ (a) study group I; (b) study group II; (c) control group.

**Table 4:** Changes in the SAS scale assessment for the GMFCS level.

GMFCS Level	Study Group I				Study Group II				Control Group				Differences between Groups *p* ^#^
	No Improvement		Improvement		No Improvement		Improvement		No Improvement		Improvement		
	(n)	(%)	(n)	(%)	(n)	(%)	(n)	(%)	(n)	(%)	(n)	(%)	
**I**	**0**	0.00	10	100.00	5	41.67	7	58.33	6	85.71	1	14.29	(a)-(b) ***p* =** 0.030 (a)-(c) ***p* =** 0.001 (b)-(c) *p* = 0.073
**II**	2	40.00	3	60.00	2	66.67	1	33.33	5	62.50	3	37.50	(a)-(b) *p* = 0.429 (a)-(c) *p* = 0.326 (b)-(c) *p* = 0.509
**level I vs. level II** ***p***	*p* = 0.095				*p* = 0.369				*p* = 0.287				

^#^ (a) study group I; (b) study group II; (c) control group.

**Table 5:** Changes in the SAS scale assessment in terms of age (for two age ranges: 6−7 and 8−12 years).

Age Ranges (Years)	Study Group I				Study Group II				Control Group				Differences between Groups *p* ^#^
	No Improvement		Improvement		No Improvement		Improvement		No Improvement		Improvement		
	(n)	(%)	(n)	(%)	(n)	(%)	(n)	(%)	(n)	(%)	(n)	(%)	
**6−7**	**0**	0.00	10	100.00	3	30.00	7	70.00	7	87.50	1	12.50	(a)-(b) *p* = 0.105 (a)-(c) ***p* =** 0.000 (b)-(c) ***p* =** 0.022
**8−12**	2	40.00	3	60.00	3	60.00	2	40.00	4	57.14	3	42.86	(a)-(b) *p* = 0.397 (a)-(c) *p* = 0.379 (b)-(c) *p* = 0.442
**6−7 years vs. 8−12 years** ***p***	*p* = 0.095				*p* = 0.240				*p* = 0.205				

^#^ (a) study group I; (b) study group II; (c) control group.

## Supplementary Materials

The following are available online at https://www.mdpi.com/1660-4601/17/18/6846/s1, Table S1: Summary of studies assessing effect of hippotherapy on children with cerebral palsy.

## References

[1] Schiariti V., Fowler E., Brandenburg J., Levey E., McIntyre S., Sukal-Moulton T., Ramey S.L., Rose J., Sienko S., Stashinko E. (2018). A common data language for clinical research studies: The National Institute of Neurological Disorders and Stroke and American Academy for Cerebral Palsy and Developmental Medicine Cerebral Palsy Common Data Elements Version 1.0 recommendations. Dev. Med. Child Neurol..

[2] Aisen M.L., Kerkovich D., Mast J., Mulroy S., Al Wren T., Kay R.M., Rethlefsen S.A. (2011). Cerebral palsy: Clinical care and neurological rehabilitation. Lancet Neurol..

[3] Morris C. (2007). Definition and classification of cerebral palsy: A historical perspective. Dev. Med. Child Neurol..

[4] Rosenbaum P., Paneth N., Leviton A., Goldstein M., Bax M. (2007). A report: The definition and classification of cerebral palsy. Dev. Med. Child Neurol..

[5] Christine C., Dolk H., Platt M.J., Colver A., Prasauskiene A., Krägeloh-Mann I., SCPE Collaborative Group (2007). Recommendations from the SCPE collaborative group for defining and classifying cerebral palsy. Dev. Med. Child Neurol..

[6] Tseng S.-H., Chen H.-C., Tam K.-W. (2012). Systematic review and meta-analysis of the effect of equine assisted activities and therapies on gross motor outcome in children with cerebral palsy. Disabil. Rehabil..

[7] Moraes A., Copetti F., Angelo V.R., Chiavoloni L.L., David A.C. (2016). The effects of hippotherapy on postural balance and functional ability in children with cerebral palsy. J. Phys. Ther. Sci..

[8] Zadnikar M., Kastrin A. (2011). Effects of hippotherapy and therapeutic horseback riding on postural control or balance in children with cerebral palsy: A meta-analysis. Dev. Med. Child Neurol..

[9] Kwon J.-Y., Chang H.J., Yi S.-H., Lee J.Y., Shin H.-Y., Kim Y.-H. (2015). Effect of Hippotherapy on Gross Motor Function in Children with Cerebral Palsy: A Randomized Controlled Trial. J. Altern. Complement. Med..

[10] Debuse D., Gibb C., Chandler C. (2009). Effects of hippotherapy on people with cerebral palsy from the users’ perspective: A qualitative study. Physiother. Theory Pr..

[11] Myhr U. (1993). Manual for the Sitting Assessment Scale.

[12] Kwon J.-Y., Chang H.J., Lee J.Y., Ha Y., Lee P.K., Kim Y. (2011). Effects of Hippotherapy on Gait Parameters in Children with Bilateral Spastic Cerebral Palsy. Arch. Phys. Med. Rehabil..

[13] Park E.S., Rha D.-W., Shin J.S., Kim S., Jung S. (2014). Effects of Hippotherapy on Gross Motor Function and Functional Performance of Children with Cerebral Palsy. Yonsei Med. J..

[14] Shurtleff T.L., Standeven J.W., Engsberg J.R. (2009). Changes in Dynamic Trunk/Head Stability and Functional Reach after Hippotherapy. Arch. Phys. Med. Rehabil..

[15] Shurtleff T., Engsberg J.R. (2010). Changes in Trunk and Head Stability in Children with Cerebral Palsy after Hippotherapy: A Pilot Study. Phys. Occup. Ther. Pediatr..

[16] Ionatamishvili N.I., Tsverava D.M., Loriya M.S., Sheshaberidze E.G., Rukhadze M.M. (2004). Riding Therapy as a Method of Rehabilitation of Children with Cerebral Palsy. Hum. Physiol..

[17] Haehl V., Giuliani C., Lewis C. (1999). Influence of Hippotherapy on the Kinematics and Functional Performance of Two Children with Cerebral Palsy. Pediatr. Phys. Ther..

[18] Bertoti D.B. (1988). Effect of therapeutic horseback riding on posture in children with cerebral palsy. Phys. Ther..

[19] MacPhail H.E.A., Edwards J., Golding J., Miller K., Mosier C., Zwiers T. (1998). Trunk Postural Reactions in Children with and Without Cerebral Palsy During Therapeutic Horseback Riding. Pediatr. Phys. Ther..

[20] Hamill D., Washington K., White O.R. (2007). The effects of hippotherapy on postural control in sitting for children with cerebral palsy. Phys. Occup. Ther. Pedi..

