# Supplementary material for: The Influence of Hippotherapy on the Body Posture in a Sitting Position among Children with Cerebral Palsy
Source: Int J Environ Res Public Health. 2020 Sep 19;17(18):6846. doi: 10.3390/ijerph17186846 (PMC7558765; doi:10.3390/ijerph17186846)
Supplement: Supplementary file 1 [file ijerph-17-06846-s001.pdf]

Suppl. Table 1. Summary of studies assessing effect of hippotherapy on children with cerebral palsy.

Study	Results
1998, Bertoti [18]	Significant improvement occurring during the period of therapeutic riding. Clinical improvements were also noted in muscle tone and balance.
1998, MacPhail et al. [19]	Horseback riding facilitate normal equilibrium reactions for children with diplegic CP.
1999, Haehl et al. [17]	Children with cerebral palsy approximated the biphasic movement patterns exhibited by the two children developing typically.
2007, Hamill et al. [20]	None of the children made gains on any of the standardized outcome measures. Parental perceptions were very positive, with reported improvements in range of motion and head control.
2009, Debuse et al. [10]	Hippotherapy improves motor skills. Users' reports indicated that hippotherapy facilitates carryover of motor ability learned during therapy session to motor activities of daily living.
2009, Shurtlett et al. [14]; 20010, Shurtlett et al. [15]	Hippotherapy improves trunk/head stability and upper extremities reaching/targeting.
2011, Kwon et al. [12]	Hippotherapy improves gait and balance in children with bilateral spastic cerebral palsy.
2014, Park et al. [13]	The beneficial effects of hippotherapy on gross motor function and functional performance in children with CP.
2015, Kwon et al. [9]	Hippotherapy positively affects gross motor function and balance in children with CP of various functional levels.
2016, Moraes et al. [7]	Hippotherapy resulted in improvement in postural balance in the sitting position, dynamic balance, and functionality in children with cerebral palsy.
